# Low-Alpha-Cellulose-Based Membranes

**DOI:** 10.3390/polym17050598

**Published:** 2025-02-24

**Authors:** Igor Makarov, Gulbarshin Shambilova, Aigul Bukanova, Fazilat Kairliyeva, Saule Bukanova, Zhanar Kadasheva, Radmir Gainutdinov, Alexander Koksharov, Ivan Komarov, Junlong Song, Sergey Legkov, Alexandra Nebesskaya

**Affiliations:** 1 A.V. Topchiev Institute of Petrochemical Synthesis RAS, Leninsky Prospect 29, 119991 Moscow, Russia; legkov@ips.ac.ru (S.L.); nebesskaya@ips.ac.ru (A.N.); 2 Department of Chemistry and Chemical Technology, Kh. Dosmukhamedov Atyrau University, Studenchesky Ave. 1, 060011 Atyrau, Kazakhstan; shambilova_gulba@mail.ru (G.S.); zh.kadasheva@asu.edu.kz (Z.K.); 3 Institute of Petrochemical Engineering and Ecology Named After N.K. Nadirov, Atyrau Oil and Gas University Named After S. Utebayev, M. Baimukhanov Street 45A, 060027 Atyrau, Kazakhstan; bukanova66@mail.ru (A.B.); kairlieva.fazi@mail.ru (F.K.); sauleshik81@mail.ru (S.B.); 4 Kurchatov Complex of Crystallography and Photonics, National Research Center “Kurchatov Institute”, Leninsky Prospect 59, 119333 Moscow, Russia; radmir@crys.ras.ru; 5 Ilim Group, 42 Ul, Dybtsyna, 165651 Koryazhma, Russia; aleksandr.koksharov@krm.ilimgroup.ru; 6 Department of Scientific Activity, Moscow Polytechnic University, St. B. Semenovskaya, 38, 107023 Moscow, Russia; master_kom@mail.ru; 7 Jiangsu Co-Innovation Center for Efficient Processing and Utilization of Forest Resources and International Innovation Center for Forest Chemicals and Materials, Nanjing Forestry University, Nanjing 210037, China; junlong.song@njfu.edu.cn

**Keywords:** cellulose, N-methylmorpholine-N-oxide, cellulosic membranes, alpha fraction, hemicellulose, morphology, permeability, transport properties, biodegradability

## Abstract

Depending on the method of cellulose production, the proportion of alpha fraction in it can vary significantly. Paper pulp, unlike dissolving cellulose, has an alpha proportion of less than 90%. The presence of cellulose satellites in the system does not impede the formation of concentrated solutions of N-methylmorpholine-N-oxide (NMMO). In the current study, spinning solutions based on cellulose with a low alpha fraction (up to 90%) (pulp cellulose) are investigated. The morphological features and rheological behavior of such solutions are examined. It is suggested to roll the obtained solutions in order to obtain cellulose membranes. X-ray diffraction, IR spectroscopy, AFM and SEM were used to investigate the resulting structure and morphology of the obtained membranes. It is shown that the degree of crystallinity for the membranes varies based on the impurity content in the sample. The morphology of the films is characterized by a dense texture and the absence of vacuoles. The highest strength and elastic modulus were found for membranes made of bleached hardwood sulfate cellulose, 5.7 MPa and 6.4 GPa, respectively. The maximum values of the contact angle (48°) were found for films with a higher proportion of lignin. The presence of lignin in the membranes leads to an increase in rejection for the anionic dyes Orange II and Remazol Brilliant Blue R.

## 1. Introduction

The most readily available natural polymer is cellulose; its raw component is constantly renewed in huge amounts [1]. The majority of cellulose is used in the production of paper, packaging materials, and derivatives [2,3,4]. The utilization of micro- and nanocellulose is in high demand for the production of stable emulsions and suspensions [5,6]. There is significant potential for employing cellulose as fillers and to create functional materials. Thus, in their study, Junlong Song et al. [7] revealed the great potential of employing colloidal systems based on disc-like CNC-II for the development of advanced materials.

The structure of native and regenerated cellulose greatly influences its distinctive properties such as high strength, hydrophilicity, sorption, chemical stability in aggressive environments, and biodegradability [8,9,10]. Because of its resilience to a variety of classic organic solvents, cellulose is suitable for the production of membranes. Along with chemical stability, cellulose membranes provide excellent mechanical properties, flexibility, hypoallergenic features, biodegradability, superior transport capabilities, and selectivity [11]. Thus, cellulose membranes can be used to purify petroleum products, binary mixes of glycerol, ethylene glycol, ethanol, formalin, acetone, and water (ultrafiltration and nanofiltration). Cellulose is not thermoplastic and, therefore, membranes are formed from it through solutions.

For the formation of cellulose solutions, it is proposed to use N_2_O_4_/DMF [12,13], DMAA/LiCl [14], aqueous solutions of zinc chloride [15], ionic liquids, aqueous solutions of NaOH and orthophosphoric acid [16,17]. Unfortunately, the high cost, regeneration difficulties, toxicity, and corrosive action of these solvents have prevented their industrial distribution. Currently, cellulose films (membranes) are made utilizing the ecologically harmful viscose process and the MMO process. In the latter procedure, N-methylmorpholine-N-oxide is utilized as a direct solvent for cellulose, dissolving up to 45% of the polymer [18].

The use of forming solutions enables control over the structure and morphology of the resultant membranes [19]. Depending on the properties of the solution and the conditions of membrane formation, they can have a porous or dense morphology, asymmetry or homogeneity along the cross-section; the use of particular polymer substrates allows for composite membranes [20,21,22,23,24,25].

The membrane structure can be changed by altering the phase inversion process during the coagulation of the spinning solution in the form of a thin film [26]. Rigid non-solvents (precipitants) cause rapid separation of the polymer phase while also forming vacuoles and defects in the formed item [27]. The use of soft precipitants delays mass transfer processes and results in a more uniform membrane morphology [28]. Water is a rigid precipitant for cellulose solutions, whereas alcohols, aqueous alcohol solutions, and solvents are soft ones [29]. Asymmetric membranes can be created by varying the intensity of the contact between the solvent and the precipitant with the transverse cleavage consisting of many layers with various structures [30]. Although the chemical composition of asymmetric membranes is constant, the porosity varies perpendicular to the membrane surface. In such membranes, the selective layer may be much thinner than the overall thickness of the membranes.

Temperature is another key component that influences the polymer phase coagulation rate [31]. By increasing it, it is possible to accelerate the penetration of the precipitant into the volume of the future membrane and form a morphology with a larger pore size. On the contrary, decreasing the temperature reduces the rate of non-solvent diffusion, allowing for a more uniform morphology with smaller pores.

Membranes derived from less concentrated solutions will have a different morphology than systems with a higher proportion of polymer phase. As a result, obtaining membranes from concentrated solutions is an important direction. Increasing the polymer concentration in the spinning solution causes a more homogenous morphology, allowing the resulting membranes to be used for nanofiltration among other applications.

Currently, most cellulose films are made by using the hazardous viscose method (cellophane). These films are widely used in a variety of applications, including food packaging and dialysis membranes. Cellulose-based films (membranes) are recognized for their biological and chemical inertness, elasticity, non-toxicity, breathability, heat resistance, and strength [32]. In recent years, a significant deal of experience has been gained in the use of cellophane to separate water- and alcohol-based systems [33,34,35]. Water and less polar liquids can pass through cellulose membranes at many times the permeability of synthetic-polymer-based membranes.

In our early work, we demonstrated that membranes produced from cellulose solutions in NMMO could serve as alternatives to cellophane [36]. The use of NMMO permits membranes to be formed from concentrated solutions containing up to 18% of cellulose. The high activity of NMMO in relation to cellulose makes it possible to obtain spinning solutions not only from dissolving pulp but also from non-traditional celluloses with low alpha fraction contents and a considerable number of contaminants (hemicellulose, inorganic compounds, etc.) [37]. In [38], it is shown that it is possible to use composite systems based on soluble cellulose and purified KRAFT lignin to obtain fibers. It is shown that the spinnability decreases with increasing lignin content. The possibility of using composite fibers based on cellulose and lignin is considered in [39]. Processing of low-refined kraft pulps into man-made fibers using ionic liquids is described in [40]. It is shown that the chemical composition and molecular integrity of the lignocellulose matrix affect spinnability. Unfortunately, in the works under consideration, the authors concentrate on the use of direct cellulose solvents for fiber formation, and cellulose membranes are ignored.

We expect that cellulose with a low alpha content (paper pulp) can also be employed to make membranes for nano- and ultrafiltration. The aim of this research was to investigate the possibility of obtaining cellulose membranes from paper pulp with a low alpha fraction content and various proportions of lignin via NMMO solutions, as well as the rheological and morphological properties of the solutions, structural features of the obtained membranes, and their transport characteristics, selectivity, and mechanical properties.

## 2. Materials and Methods

### 2.1. Materials

To produce cellulose films with low alpha fraction contents, cellulose from the Kotlas Pulp and Paper Mill (Ilim Group, Koryazhma, Russia) was utilized. The characteristics of the following pulps are presented in Table 1:

(1) Bleached sulphate hardwood pulp grade “NS-Extra” with the following composition: 30% birch and 70% aspen (Cell 1);
(2) Unbleached sulphate softwood pulp grade “Extra” with the following composition: 80% spruce and 20% pine (Cell 2);
(3) Unbleached sulphate softwood pulp grade “Fiber” with the following composition: 80% spruce and 20% pine (Cell 3).

The equilibrium moisture content of cellulose was estimated according to GOST 16932-93 (ISO 638-78). GOST 6840-78 (TAPPI. 211 om-02) [41] was used to determine the proportion of alpha fraction.

N-methylmorpholine-N-oxide with a melting point of 120–130 °C (water content of 8–10 wt.%) supplied by Demochem (Shanghai, China) was used as a solvent for cellulose. To suppress thermal oxidation, 0.5 wt.% propyl gallate (Sigma-Aldrich, St. Louis, MI, USA) was introduced into the solutions.

### 2.2. Formation of Membranes

**Figure 1:**
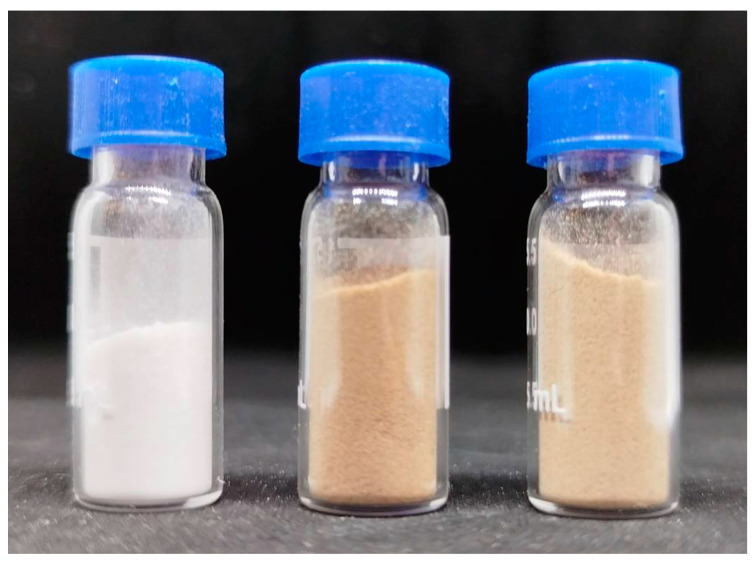
Photograph of cellulose powders. Cell 1 (bleached sulphate hardwood pulp grade “NS-Extra”), Cell 2 (unbleached sulphate softwood pulp grade “Extra”), and Cell 3 (unbleached sulphate softwood pulp grade “Fiber”) (from left to right).

To prepare solutions of cellulose in NMMO, the “solid-phase dissolution” method was used [42]. The procedure consists of the following steps:

1. Cellulose sheets are crushed to a powder state with a particle size of up to 200 microns (Figure 1).
2. Cellulose, propyl gallate and NMMO powders are mixed in the ratio required to obtain 16% solutions.
3. The system is mechanically activated through intense shear deformation in a compressed state. This leads to the formation of H-complexes.
4. The solution is transferred to a molten state and mixed at a temperature of up to 110–120 °C, using a laboratory twin-screw extruder from Haake Minilab II (ThermoFisher Scientific, Dreieich, Germany).

### 2.3. Morphology and Rheology of Solutions

The quality of the cellulose solutions was evaluated using a polarization microscope with a heated stage (microscopy “Boetius”, VEB Kombinat Nadema, Ruhla, former GDR).

The viscosity of solutions was determined using a HAAKE MARS 60 rotational rheometer (ThermoFisher Scientific, Dreieich, Germany) with the cone–plane unit, a diameter of 20 mm, and an angle of 1° under conditions of continuous deformation in a shear rate range of 10^−3^ to 10^3^ s^−1^. To prevent drying of the sample, the sensor system was filled with PMS-100 silicone oil (JSC Silan, Dankov, Russia).

### 2.4. Structure and Chemical Composition of Membranes

The structure of the cellulose membrane was studied using X-ray diffractometry with a Rigaku Rotaflex D/MAX-RC setup equipped with a rotating copper anode (X-ray source operating mode 30 kV, 100 mA, characteristic radiation wavelength λ = 0.1542 nm, CuKβ radiation absorbed by a nickel filter), a horizontal goniometer, and a scintillation detector. X-ray experiments were performed in the reflection mode according to the Bragg–Brentano scheme in the continuous θ–2θ scanning mode in the angular range of 2.5–50.0° and with a scanning step of 0.04° at room temperature.

The morphology of the membrane transverse cleavages was studied using low-voltage scanning electron microscopy (SEM) on an FEI Scios microscope (Hillsboro, OR, USA) with an accelerating voltage of less than 1 kV in the secondary electron mode. To produce SEM micrographs of cross-sections, cellulose membranes were pre-cooled in liquid nitrogen before being cut with a knife.

The IR spectra of the membranes were recorded using a HYPERION-2000 IR microscope and an IFS-66 v/s Bruker IR Fourier spectrometer (crystal–Ge, scan. 50, resolution 2 cm^−1^, range 4000–600 cm^−1^).

The surface topography of the cellulose films was studied using an NTEGRA PRIMA SPM atomic force microscope (AFM) (NT-MDT, Moscow, Russian Federation) in tapping mode with an HA_FM cantilever (beam B) (Tipsnano OÜ, Tallinn, Estonia) with a resonance frequency of f = 114 kHz, force constant of k = 6 N/m and tip radius of R < 10 nm.

Chemical analysis was conducted using a Shimadzu ICPE-9000 emission inductively coupled plasma spectrometer (Shimadzu, Kyoto, Japan). Mineralization was carried out on a TOREX+ microwave sample preparation system (PreeKem). The samples to be tested were placed in KJ-160 fluoroplastic containers and treated with 5 mL of HNO_3_. The collected containers were then sent to the microwave system chamber.

### 2.5. Membranes Properties

To assess the transport properties of cellulose membranes, ethanol was used with a water content of 4 wt%. Rejection was estimated based on two anionic dyes, Orange II (Sigma Aldrich, Hamburg, Germany) and Remazol Brilliant Blue R (Sigma Aldrich, Darmstadt, Germany), with molecular weights of 350 and 626 g/mol, respectively. Orange II (Remazol Brilliant Blue R) dye solutions in ethanol at concentrations of 30 mg/L were produced. All reagents were chemically pure and used without additional purification.

The nanofiltration characteristics of cellulose membranes were studied in stainless-steel dead-end filter cells equipped with magnetic stirrers at a membrane pressure of 15 bar, as described earlier. The effective membrane area was 7.9 cm^2^. The volume of the liquid under study was selected so that no more than 20% of the membrane volume fell on the membrane during the experiment. The pressure in the filter cell was provided by helium.

The rejection of the membrane (R) was determined based on the optical density of the liquid in the cell (A_f_) and permeate (A_p_):

(1) $$R=\left(1-\frac{A_{p}}{A_{f}}\right)100\%$$

According to Behr’s law, the value of A is directly proportional to the concentration of the component, which makes it possible to use optical density instead of concentration in Formula (1). Spectra of dye solutions were taken in the visible and UV regions to assess the rejection of membranes. Optical density was measured using a PE-5400UF spectrophotometer with water as the reference solution. The concentrations of the model dyes Remazol Brilliant Blue R and Orange II were determined to be λ = 592 nm and λ = 483 nm, respectively.

### 2.6. Mechanical Properties

The mechanical properties of the undried and dry membranes were evaluated using an Instron 1122 tensile testing machine (Instron, Norwood, MA, USA). The membrane deformation rate was 10 mm/min. The films for tensile testing were cut into strips. The initial distance between grips was 10 mm.

### 2.7. Contact Angle

Contact angle measurements were obtained using a self-designed measurement system containing an optical microscope with a 3.5× lens and a 5 MP CMOS digital camera (PRC). Photographs of contact angles were obtained using the ImageView software version 4.8 supplied with the digital camera. Drying time was measured using the time scale in the ImageView program. Data processing and contact angle measurements were carried out using the “contact angle” plugin in ImageJ open-source software (USA, https://imagej.net (accessed on 10 November 2024)). A mechanical 0.1–2.5 µL pipette (BIOHIT Proline, Goettingen, Germany) was used to apply the drop; the volume of the applied drop was 0.4 μL.

Drying time occurs from the moment the drops are applied until the visual drying of the drop in the lens of the contact angle meter and the sporting effect on the surface of the substrate. When recording a video file, filming was slowed down by 16 times due to the rapid drying of a drop of ethanol. Time measurement was carried out according to the time scale of the video file, considering the filming slowdown factor. Data processing and determination of the contact angle were carried out using ImageJ open-source software (USA, https://imagej.net (accessed on 10 November 2024)).

## 3. Results and Discussion

It is well known that in order to achieve stable cellulose solutions in NMMO, a polymer with high purity should be used. First of all, when pulping cellulose, emphasis is devoted to the removal of hemicellulose, lignin, and inorganic compounds (e.g., metals, such as iron). Table 2 shows the elemental makeup of the cellulose samples under investigation. The iron content in soluble cellulose should not exceed 8 ppm [43].

Ag, Au, Ba, Cd, Co, Cr, Cu, La, Li, Mo, Na, Ni, Pb, Pd, Pt, Re, Rh, Ru, Sn, Ti, and V are not confirmed in the samples (below the detection limit). Unbleached cellulose has more contaminants than bleached cellulose. As a result, the calcium level in the Cell 3 sample is nearly an order of magnitude higher than in Cell 1. Cell 2 contains around 152 ppm of magnesium, while Cell 1 contains 38 ppm. All samples have an iron concentration of less than 5.5 ppm, which meets the standards for soluble cellulose. That is, the samples in question can be used to produce spinning solutions.

**Figure 2:**
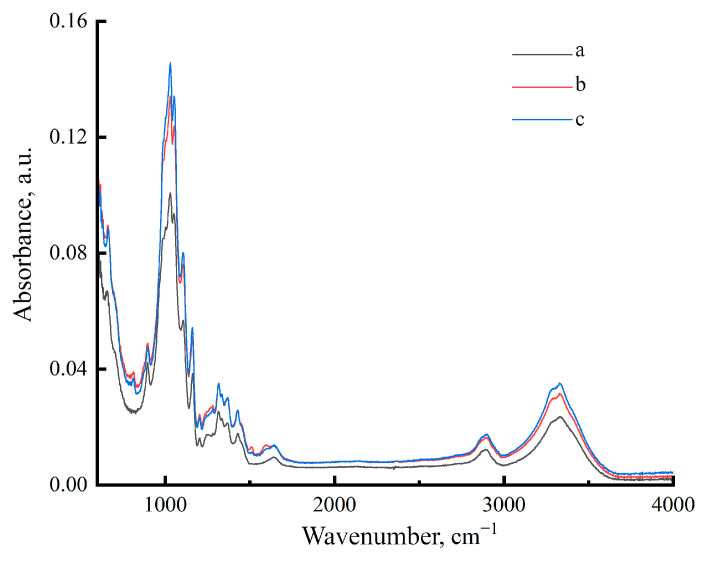
IR spectra of cellulose powders Cell 1 (a), Cell 2 (b) and Cell 3 (c).

Figure 2 shows the FTIR spectra of Cell 1, Cell 2 and Cell 3 in the range of 4000–600 cm^−1^.

Since the cellulose employed in this study did not have its hydrogen bond system broken during regeneration, the results should be similar to the spectra of native cellulose (polymorph I) [44]. For cellulose, a number of major bands are recognized that characterize the functional groups, and bonds and are also used to measure the polymer supramolecular ordering level. The band at 898 cm^−1^ characterizes the 1,4-glycosidic bond and can be used to determine the presence of an amorphous phase in cellulose. When describing OH groups, the bands at 1335 cm^−1^ (OH), 1430 cm^−1^ deformation in-plane vibrations of the OH group, 1620–1641 cm^−1^ (OH) for adsorbed water, and 3100–3600 cm^−1^ (OH) are relied upon. The bands at 1107 and 1161 cm^−1^ correspond to asymmetric stretching vibrations of the C–O–C bridge, 1315 cm^−1^ (CH_2_), and 2850 cm^−1^ (CH).

The presence of impurities and varied cellulose structures causes the samples under study to have varying quantities of adsorbed moisture. For sample Cell 2, the intensity of the 1637 cm^−1^ band is the highest, which confirms the presence of a larger amount of moisture in cellulose. The lowest intensity of this band was observed for sample Cell 1.

In the absorption bands at 1512 cm^−1^ (the skeletal vibrations of the aromatic ring) and 1600 cm^−1^ (aromatic skeletal vibration) spectra have different intensities. Based on these bands, one can say which sample contains a greater amount of lignin [45]. For the Cell 2 sample, the highest intensity is observed, which indicates a higher lignin content, for bleached cellulose; on the contrary, the intensity is the lowest, and the proportion of lignin is correspondingly lower.

The structural characteristics of the samples used can be estimated based on the bands at 898 cm^−1^ (the amorphous phase) and 1428–1430 cm^−1^ (crystalline phase) [44,46] and the intensity ratio of these bands: I_1430_/I_898_ [47]. The crystallinity index for Cell 1 is 0.42; for unbleached samples Cell 2 and Cell 3, it is 0.52 and 0.54, respectively. Thus, bleached cellulose has a more disordered structure compared to unbleached cellulose samples.

**Figure 3:**
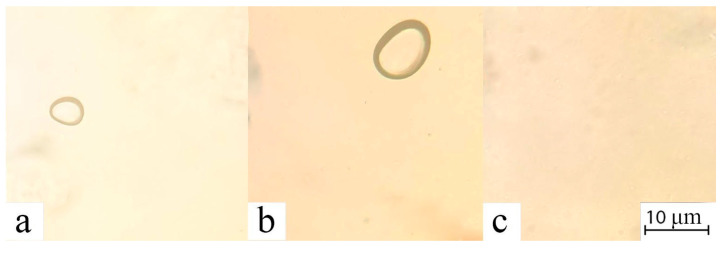
Morphology of cellulose solutions: Cell 1 (**a**), Cell 2 (**b**) and Cell 3 (**c**) in NMMO (oval inclusions—air).

Despite the presence of impurities in cellulose, all samples dissolve readily in NMMO, forming homogenous solutions. Figure 3 shows the morphology of the resulting solutions.

**Figure 4:**
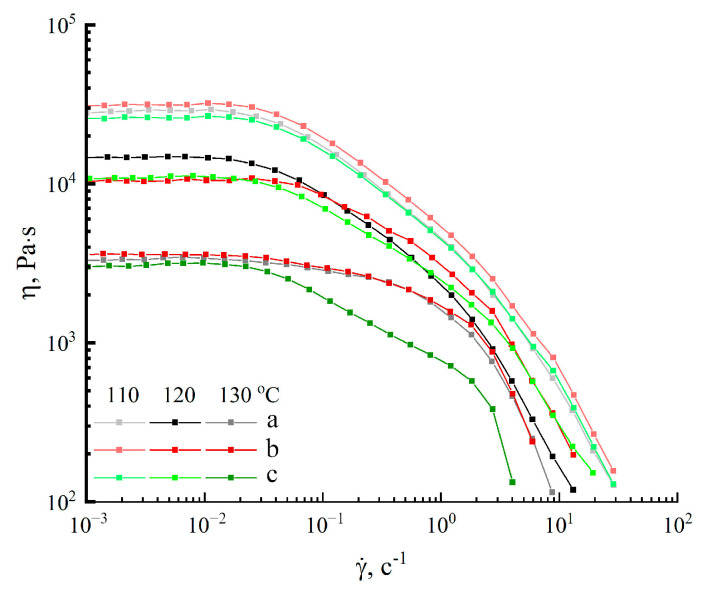
Flow curves of 18% cellulose solutions for Cell 1 (a), Cell 2 (b) and Cell 3 (c) in NMMO at different temperatures.

The solutions produced are transparent in transmitted light and do not glow in crossed polaroids. The color of the solutions varies from light yellow for bleached to light brown for unbleached cellulose. The homogeneity of the resultant solutions demonstrates the absence of undissolved residues, and the solutions can be regarded as spinning. Before the formation of membranes, the rheological behavior of the solutions was evaluated. First of all, the viscosities of cellulose solutions Cell 1, Cell 2, and Cell 3 were evaluated at different temperatures (Figure 4).

Based on past expertise in obtaining solutions for membrane production, 18% cellulose solutions were developed. For these, the steady-state viscosity (η)–shear rate (γ) dependence was obtained at temperatures ranging from 110 to 130 °C. At low shear rates, all solutions fall into the Newtonian zone. However, only in the Cell 1 sample does this region span two decades of shear rates. Higher shear rates cause a decrease in viscosity (shear thinning), which is associated with the destruction of solution structure. The viscosity of the solutions decreases with increasing temperature, as is expected for polymer solutions. Thus, heating the systems from 110 to 130 °C reduced their viscosity by nearly an order of magnitude. The opposing process of cooling the system results in more dramatic shear-thinning, and the Newtonian area shifts somewhat to a lower shear rate region. That is, the observed processes of solution structural reorganization and macromolecule orientation along the deformation axis begin sooner at lower temperatures [48]. Despite the varied component composition of cellulose following mechanical milling and solution formation, changes in viscosity characteristics are almost non-existent.

**Figure 5:**
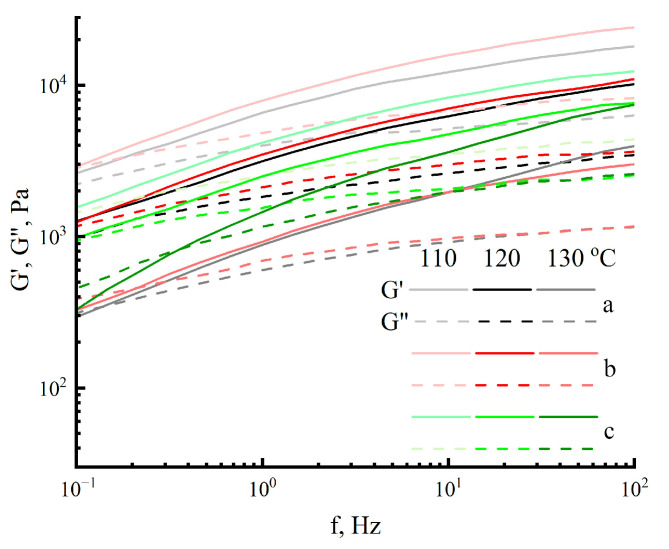
G′ and G″ as a function of oscillatory frequency (18% cellulose solutions in NMMO: Cell 1 (a), Cell 2 (b) and Cell 3 (c)).

Figure 5 shows the typical frequency sweep measurements for cellulose solutions.

Most curves have a higher elastic modulus (G′) than loss modulus (G″), indicating that elastic properties are more dominant than viscous ones. As the deformation frequency increases, the values of G′ rise faster than G″. The Cell 2 cellulose solution exhibits the highest elastic modulus values at 110 °C. The crossover point (equivalent module values) is only observed in solutions at 130 °C. At lower temperatures and lower deformation frequencies, the crossover point is likely to be outside the measurement range. That is, all of the examined cellulose solutions are viscoelastic liquids.

**Figure 6:**
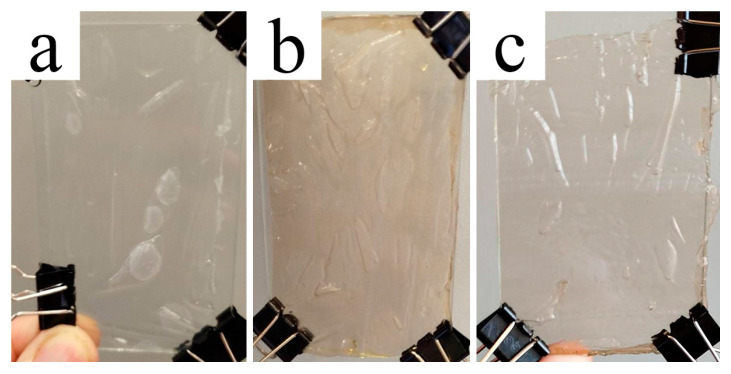
Photographs of cellulose films Cell 1 (**a**), Cell 2 (**b**) and Cell 3 (**c**).

The membranes were made from 18% cellulose solutions using the rolling method, which allows for membranes with sufficient width and length. The use of anti-adhesive coating films enables a small layer of solution to pass through the rollers without adhering or causing defects. Figure 6 depicts photographs of the films obtained.

**Figure 7:**
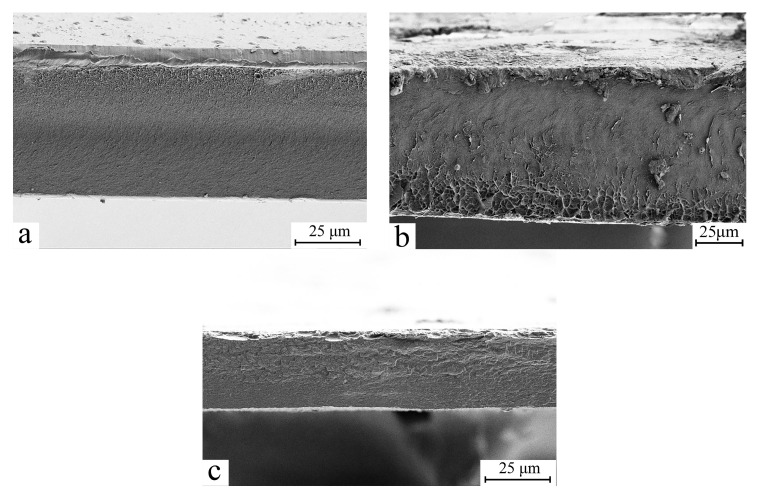
SEM micrographs of cellulose membranes Cell 1 (**a**), Cell 2 (**b**) and Cell 3 (**c**).

As seen in the figure, the color differences between the original samples remain even after solutions and films are formed. Despite the color variation, the finished films are clear. Cell 1 cellulose samples exhibit almost minimal yellowness, while Cell 2 yields the most intense hue. The transparency of the films suggests a homogeneous shape. Figure 7 depicts micrographs of transverse cleavages in cellulose films.

All three films have a similar morphology. The analysis of SEM images reveals that coagulation of just-formed membranes in a rigid aqueous precipitant did not result in the creation of vacuoles and big pores typical of such coagulation. At low magnifications, transverse chips show no significant asymmetry. A more comprehensive study of the films reveals a thin surface layer with a denser structure. The thickness of this layer is limited to a few microns. This layer was most likely formed as a result of the extreme precipitation circumstances encountered when using water. As water penetrates the membrane volume, the precipitant “softens” with the solvent, and the polymer phase is released under other conditions that lead to the production of an excellent morphology, with all membranes exhibiting no microvoids.

**Figure 8:**
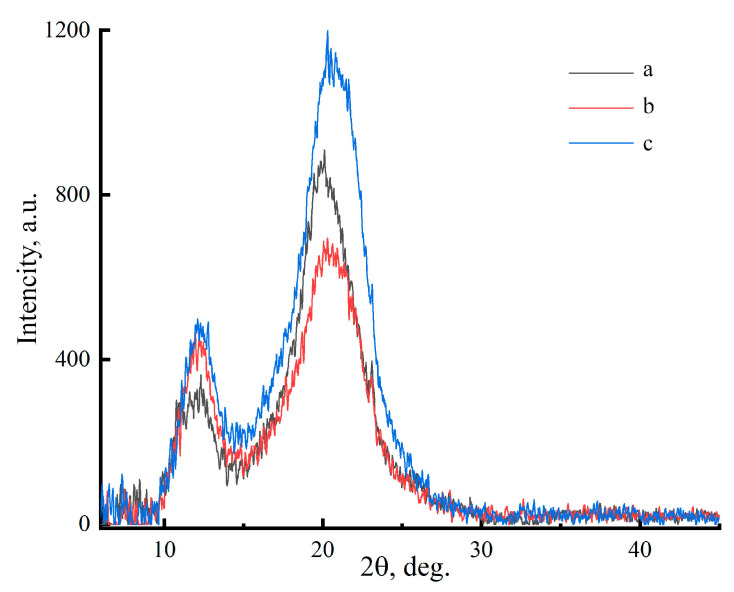
Diffraction patterns of cellulose membranes Cell 1 (a), Cell 2 (b) and Cell 3 (c).

The influence of the type of cellulose on the forming structure of cellulose is shown in Figure 8.

The precipitant interacts with the solution and separates the cellulose phase. When the solvent is removed, intermolecular hydrogen bonds are formed in the cellulose. The regenerated membranes have a diffraction pattern that corresponds to cellulose polymorph II. The main reflections on the equatorial diffraction patterns are in the regions of 2θ~12.1°, ~20.1° and ~21.5° [49,50]. The crystallinity of the obtained films is practically independent of the history of cellulose production; the lowest values were observed for sample Cell 1, and the highest values were observed for Cell 3. As for the crystallite sizes, their values coincide for samples Cell 1 and Cell 2 and are 2.8 nm, respectively; for sample Cell 3, the obtained values are smaller and equal to 2.7 nm.

**Figure 9:**
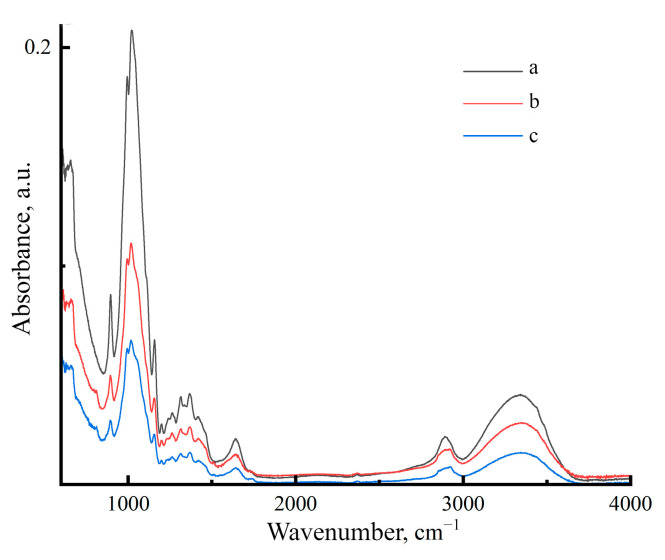
IR spectrum of membranes Cell 1 (a), Cell 2 (b) and Cell 3 (c).

Figure 9 presents the FTIR spectrum of the samples after dissolution and regeneration.

It is well known that after cellulose regeneration, a new system of hydrogen bonds is formed, which corresponds to the spectra presented above. The characteristic bands for regenerated cellulose are in the spectral region of 1500–850 cm^−1^ [51]. In his work, O’Connor emphasizes how structural changes in cellulose are mirrored in the intensity and position of cellulose I’s characteristic bands. Of particular note is the region of 3600–3100 cm^−1^ (OH), for which the intensity of the bands is noticeably reduced. In work [52], for cellulose II, it is proposed to rely on the 3439 and 3342 cm^−1^ bands.

The obtained spectra were used to evaluate the total crystalline index (TCI) (I_1378_/I_2900_), lateral order index (LOI) (I_1437_/I_899_) and hydrogen bond intensity (HBI) (I_3336_/I_1336_).

Table 3 shows that the history of obtaining cellulose and membranes from its solutions is reflected in the HBI, LOI, and TCI indices. Samples with a high lignin concentration exhibit the most hydrogen bonding. The number of hydrogen bonds in bleached cellulose is the smallest, with an HBI value of 1.12. As the HBI increases, so do the LOI values. For the TCI, the lowest values were reported for the Cell 2 sample with the largest proportion of lignin, indicating a reduction in crystallinity as the proportion of amorphous to crystalline region increases. In samples with a higher proportion of hemicellulose and a lower lignin concentration, the TCI increases as expected.

The degree of order in the system is determined by the LOI, which is defined as the ratio of the intensities of the bands characteristic of the crystalline and amorphous phases. A high LOI value indicates tight packing of cellulose macromolecules and a decreased amorphous phase volume [53]. For the examined samples, the LOI values increase as the amount of lignin increases.

**Figure 10:**
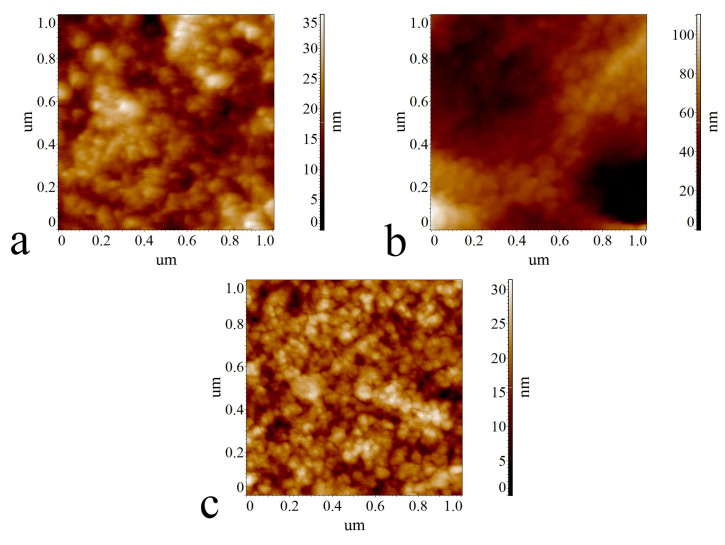
AFM images for membrane samples Cell 1 (**a**), Cell 2 (**b**) and Cell 3 (**c**).

Analysis of AFM images of the surface of the Cell 1 sample with a large size (20 × 20 μm, 10 × 10 μm and 5 × 5 μm) showed that the surface of the sample is smooth with a small number of rounded depressions characterized be a diameter of 200 nm to 2000 nm and a depth of 80 to 500 nm. The depressions have a slight orientation. A small number of oriented particles with a width of 100 nm to 500 nm and a length of 150 nm to 1000 nm are also observed. Analysis of the images (Figure 10) of the surface of smaller sizes (2 × 2 μm, 1 × 1 μm, 500 × 500 nm and 200 × 200 nm) showed that the surface has a uniform globular structure consisting of rounded and elongated particles with a width of 10 nm to 50 nm and a length of 20 nm to 100 nm with smaller particles predominating. It should be noted that the surface of the Cell 1 sample has the least developed relief (the roughness parameters for the 20 × 20 μm and 10 × 10 μm images were Ra = 27.80 nm, Rq = 43.50 nm and Ra = 10.50 nm, Rq = 15.70 nm, respectively).

Analysis of large-size (20 × 20 μm, 10 × 10 μm and 5 × 5 μm) Cell 2 sample surface images showed that the sample surface has the most developed relief (roughness parameters for 20 × 20 μm and 10 × 10 μm images were Ra = 322 nm, Rq = 395 nm and Ra = 184 nm, Rq = 226 nm, respectively).

The values of these parameters are an order of magnitude higher than the values of the roughness parameters of the images of the equivalent sizes of the Cell 1 sample. From the images of the surface of the large-sized Cell 2 sample (20 × 20 μm, 10 × 10 μm and 5 × 5 μm), it is evident that the surface relief is highly non-uniform with extended branched dendrite-like formations observed, the width of which ranges from 150–200 nm to 2500–2800 nm and the length from 400–800 nm to 10 μm–20 μm and possibly more (maximum image size 20 × 20 μm). Rounded particles can be seen near the branching nodes of the formation. Analysis of images of the surface of smaller sizes between the strands (filaments) (2 × 2 µm, 1 × 1 µm, 500 × 500 nm and 200 × 200 nm) showed that the surface has a less even, wavy relief but also has a uniform globular structure consisting of rounded and elongated particles with a width of 20–30 nm to 50–70 nm and a length of 30 nm to 100 nm. Thus, the nanostructures of the surfaces of samples Cell 1 and Cell 2 are similar, although the surface of sample Cell 2 is characterized by a slightly larger average particle size with a smaller aspect ratio.

Analysis of large-sized (20 × 20 μm, 10 × 10 μm and 5 × 5 μm) surface images of the Cell 3 sample showed that the sample surface occupies an intermediate position between Cell 1 and Cell 2 samples in terms of the degree of relief development (the roughness parameters for 20 × 20 μm and 10 × 10 μm images were Ra = 42.80 nm, Rq = 55.50 nm and Ra = 24.40 nm, Rq = 31.70 nm, respectively). The values of these parameters are closer to those of the Cell 1 sample. On the surface, there is a smaller number of non-branching strands (filaments) than in the Cell 2 sample, formed from round particles with a diameter from 80–120 nm to 300–500 nm. The length of the filaments can vary from 700 to 1000 nm to 20 µm or more (maximum image size 20 × 20 µm).

Analysis of the images of the surface of smaller sizes between the strands (filaments) (2 × 2 μm, 1 × 1 μm, 500 × 500 nm and 200 × 200 nm) revealed significant differences compared to samples Cell 1 and Cell 2. The surface of sample Cell 3 has a wavy but smooth relief, i.e., no globular structure is observed; only individual rounded particles with diameters of 15–50 nm and 80–120 nm appear to be present.

When dried, the resulting cellulose membranes undergo structural changes. When water is withdrawn, some of the pores collapse, causing the morphology that formed during precipitation to shift dramatically. During subsequent wetting and expansion of the membrane, the collapsed pores are not always restored. That is, drying the membrane and modifying its structure is an irreversible process. Therefore, in some cases, to preserve the formed structure, cellulose membranes are not dried, but are instead stored in a preservative liquid. The mechanical properties for never-dried cellulose membranes are presented in Table 4.

The wet membrane thickness was measured by putting the samples between two cover glasses and using an electron micrometer. The obtained values varied from 74 to 116 μm. The Cell 1 sample had the maximum strength since all of the lignin had been removed. At a high lignin level (sample Cell 2), the strength of the films decreased by more than 45%. The strength of the Cell 3 membrane, which contained 5.1% lignin, was higher than that of the Cell 2 membrane but lower than of the Cell 1 membrane. The elastic modulus of the membranes decreased when the lignin content increased from Cell 1 to Cell 2. The Cell 2 membranes had the lowest relative elongation values, which was likely related to their high lignin concentration.

The mechanical properties of membranes dried in room conditions to equilibrium moisture content are presented in Table 5.

Drying Cell 1 membranes results in higher strength and modulus values compared to samples with high lignin concentrations. In contrast, the relative elongation values decline and are slightly lower than in the Cell 2 sample. The composition of the original cellulose impacts the mechanical properties of the membranes and provides necessary indicators.

**Figure 11:**
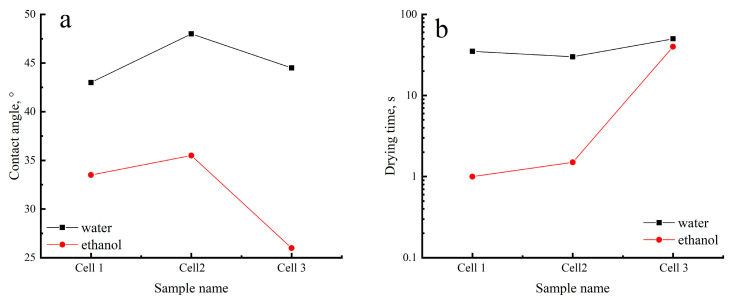
Contact angle (**a**) and drying time (**b**) of cellulose samples.

Figure 11 indicates a small increase in contact angle for both water and ethanol in the case of Cell 2. In Cell 3, however, we see a decrease in contact angle, indicating improved wettability for both water and ethanol; lowering the wetting angle has a particularly strong influence on ethanol. Water contact angle values correlate with the LOI parameter that characterizes the degree of orderliness. So, we observe an almost linear correlation: the higher the LOI, the higher the contact angle, which means that wettability increases with the increase in orderliness.

On the contrary, the drying time for Cell 3 is significantly higher both in the case of water and ethanol. We attribute this effect to the structural features of the membrane and the presence of impurities in it.

The obtained contact angle values correlate with the literature data. For example, in [54], the water contact angle for unmodified cellulose nanofiber is 13 ± 2°, which is significantly lower than that observed in the present work, due to the different origin of the fibers. In work [55], the water contact angle of the initial cellulose sponge is about 45°, which correlates well with the results of our work. In [56], water contact angles of approximately 48–50° were observed for regenerated cellulose film deposited using spin-coating. Moreover, in [57], water contact angles of about 45° were observed for nanocellulose films prepared by compressing cellulose nanocrystal pellets.

The revealed values of the wetting angle and drying time reflect the influence of cellulose impurities in the membranes, as well as the structure formed in them. The following question arises: how will the residual lignin affect the filtration properties of the membranes? The obtained values are presented in Table 6.

The table indicates that increasing the lignin content of cellulose membranes increases the rejection values for both dyes. The highest results are achieved with Cell 3 membranes. It is important that these samples include not only lignin but also a higher level of Fe than bleached cellulose. The formed structure allows for a retention of 39.7% for Remazol Brilliant Blue R and 20.1% for Orange II. The results are lower than those previously reported for cellophane [36]. Thus, for the first time, it has been demonstrated that modifying the proportion of cellulose satellites in the initial pulp allows for the formation of membranes with different structures and retention capacities to Remazol Brilliant Blue R and Orange II.

Thus, for the first time, cellulose membranes have been obtained from solutions in NMMO through a solid-phase activation stage using cellulose with a low alpha fraction content. The resulting films have good mechanical and functional properties, allowing them to be considered as potential alternatives to cellophane, which is manufactured using an environmentally hazardous process.

## 4. Conclusions

To obtain spinning solutions of cellulose for the formation of fibers, it is recommended to use highly pure soluble cellulose. This is due to the fact that the solutions must be stable and have good formability when extruded through a capillary. In the case of films, the forming conditions are different. It has been shown that the rolling method can be used to obtain cellulose membranes from cellulose solutions in NMMO. The concentrated solutions themselves can be obtained not only from soluble cellulose but also from raw materials with a high proportion of impurities. The presence of impurities in the resulting films affects the resulting structure and properties of the membranes. It has been shown that residual lignin, which often includes iron impurities, in cellulose affects the selective properties of the membranes, significantly increasing the rejection coefficient. The resulting membranes have good mechanical properties and can be used to remove dyes from a number of solvents.

## Figures and Tables

**Table 1:** Characteristics of cellulose.

	Cell 1	Cell 2	Cell 3
Viscosity (ISO 5251-2010), mL/g	810	1090	995
Lignin, %	0	8.1	5.1
Ash, %	0.093	0.115	0.171
Fiber length, mm	1.05	3.05	2.95
α-cellulose, %	87	89	88
Water, %	5.73	6.57	6.52

**Table 2:** Content of metals in samples, ppm.

Element	Cell 1	Cell 2	Cell 3
Al	2.58	10.17	4.28
Ca	191.69	1109.38	1118.77
Fe	3.87	3.63	5.45
K	≤DL *	≤DL *	203.27
Mg	38.34	151.89	121.11
Mn	2.90	61.05	58.80
Zn	≤DL *	4.00	10.90

* Detection limit.

**Table 3:** Total crystalline index (TCI), lateral order index (LOI) and hydrogen bond intensity (HBI) obtained from IR analysis of the cellulose membranes.

Sample	TCI	LOI	HBI
Cell 1	1.81 ± 0.02	0.34 ± 0.012	1.12 ± 0.01
Cell 2	1.56 ± 0.05	0.41 ± 0.026	1.20 ± 0.02
Cell 3	1.83 ± 0.03	0.36 ± 0.022	1.14 ± 0.01

**Table 4:** Mechanical properties of membranes that were not dried.

Membrane	Thickness, µm	Tensile Strength, MPa	Elongation at Break, %	Young Modulus, GPa
Cell 1	74–92	3.8–5.7	147–169	4.7–6.4
Cell 2	86–92	1.3–3	105–143	2.8–5.1
Cell 3	87–116	3.5–3.9	146–175	4.3–5.7

**Table 5:** Mechanical properties of dry membranes.

Membrane	Thickness, µm	Tensile Strength, MPa	Elongation at Break, %	Young Modulus, GPa
Cell 1	30–50	67–83	4.2–13.5	1031–1937
Cell 2	51–64	34–53	6.3–16.7	830–1142
Cell 3	38–51	41–62	6.5–12.2	1047–1242

**Table 6:** Rejection coefficient (R) values of Remazol and Orange II dyes for cellulose-based membranes.

Membrane	R_Remazol_, %	R_Orange_, %
Cell 1	5.6	2.9
Cell 2	19.7	18.7
Cell 3	39.7	20.1

## Data Availability

The original contributions presented in the study are included in the article; further inquiries can be directed to the corresponding author.

## References

[1] Gismatulina Y.A., Budaeva V.V., Veprev S.G., Sakovich G.V., Shumny V.K. (2015). Cellulose from various parts of Soranovskii Miscanthus. Russ. J. Genet. Appl. Res..

[2] Irimia A., Grigoraș V.C., Popescu C.-M. (2024). Active Cellulose-Based Food Packaging and Its Use on Foodstuff. Polymers.

[3] Seddiqi H., Oliaei E., Honarkar H., Jin J., Geonzon L.C., Bacabac R.G., Klein-Nulend J. (2021). Cellulose and its derivatives: Towards biomedical applications. Cellulose.

[4] Wang Q., Zhang X., Tian J., Zheng C., Khan M.R., Guo J., Zhu W., Jin Y., Xiao H., Song J. (2023). High throughput disassembly of cellulose nanoribbons and colloidal stabilization of gel-like Pickering emulsions. Carbohydr. Polym..

[5] Vinogradov M.I., Makarov I.S., Golova L.K., Gromovykh P.S., Kulichikhin V.G. (2020). Rheological Properties of Aqueous Dispersions of Bacterial Cellulose. Processes.

[6] Li J., Wang Z., Wang P., Tian J., Liu T., Guo J., Zhu W., Khan M.R., Xiao H., Song J. (2024). Effects of hydrolysis conditions on the morphology of cellulose II nanocrystals (CNC-II) derived from mercerized microcrystalline cellulose. Int. J. Biol. Macromol..

[7] Nishiyama Y., Langan P., Chanzy H. (2002). Crystal Structure and Hydrogen-Bonding System in Cellulose Iβ from Synchrotron X-ray and Neutron Fiber Diffraction. J. Am. Chem. Soc..

[8] Gardner K.H., Blackwell J. (1974). Hydrogen Bonding in Native Cellulose. Biochim. Biophys. Acta.

[9] Poletto M., Ornaghi H.L.J., Zattera A.J. (2014). Native Cellulose: Structure, Characterization and Thermal Properties. Materials.

[10] Makarov I.S., Shambilova G.K., Vinogradov M.I., Anokhina T.S., Bukanova A.S., Kairliyeva F.B., Bukanova S.K., Levin I.S. (2023). Membranes Based on Cellulose and Copolymers of Acrylonitrile Prepared from Joint Solutions. Membranes.

[11] Pavlyuchenko M.M., Kaputsky F.N., Grinshpan D.D. (1975). Effect of organic solvent nature on the interaction of cellulose with nitrogen tetroxide. J. Appl. Chem..

[12] Hammer R.B., Turbak A.F. (1977). Production of Rayon from Solutions of Cellulose in N_2_O_4_-DMF in Solvent Spun Rayon, Modified Cellulose Fibers and Derivatives.

[13] Yudianti R., Syampurwadi A., Onggo H., Karina M., Uyama H., Azuma J. (2016). Properties of bacterial cellulose transparent film regenerated from dimethylacetamide–LiCl solution. Polym. Adv. Technol..

[14] Lu X., Shen X. (2011). Solubility of bacteria cellulose in zinc chloride aqueous solutions. Carbohydr. Polym..

[15] Grinshpan D.D., Gonchar A.N., Tsygankova N.G., Makarevich S.E., Savitskaya T.A., Sheimo E.V. (2011). Rheological properties of concentrated solutions of cellulose and its mixtures with other polymers in orthophosphoric acid. J. Eng. Phys. Thermophys..

[16] Boerstoel H., Maatman H., Westerink J.B., Koenders B.M. (2001). Liquid crystalline solutions of cellulose in phosphoric acid. Polymer.

[17] Golova L.K., Romanov V.V., Lunina O.B., Platonov V.A., Papkov S.P., Khorozova O.D., Yakshin V.V., Belasheva T.P., Sokira A.N. (1991). Method for Obtaining a Solution for Spinning. Fibers. Patent.

[18] Grzybek P., Dudek G., van der Bruggen B. (2024). Cellulose-based films and membranes: A comprehensive review on preparation and applications. J. Chem. Eng..

[19] Cazón P., Vázquez M., Velazquez G. (2018). Novel composite films based on cellulose reinforced with chitosan and polyvinyl alcohol: Effect on mechanical properties and water vapour permeability. Polym. Test..

[20] Wu Q., Wu B. (1995). Study of membrane morphology by image analysis of electron micrographs. J. Membr. Sci..

[21] Zare F., Gonçalves S.B., Faria M., Gonçalves M.C. (2023). Improving Structural Homogeneity, Hydraulic Permeability, and Mechanical Performance of Asymmetric Monophasic Cellulose Acetate/Silica Membranes: Spinodal Decomposition Mix. Membranes.

[22] Shen L., Cheng R., Yi M., Hung W.-S., Japip S., Tian L., Zhang X., Jiang S., Li S., Wang Y. (2022). Polyamide-based membranes with structural homogeneity for ultrafast molecular sieving. Nat. Commun..

[23] Liang Y., Zhu Y., Liu C., Lee K.-R., Hung W.-S., Wang Z., Li Y., Elimelech M., Jin J., Lin S. (2020). Polyamide nanofiltration membrane with highly uniform sub-nanometre pores for sub-1 Å precision separation. Nat. Commun..

[24] Liang S., Zhang L., Li Y., Xu J. (2007). Fabrication and Properties of Cellulose Hydrated Membrane with Unique Structure. Macromol. Chem. Phys..

[25] Yushkin A.A., Anokhina T.S., Volkov A.V. (2015). Application of cellophane films as nanofiltration membranes. Pet. Chem..

[26] Durmaz E.N., Çulfaz-Emecen P.Z. (2018). Cellulose-based membranes via phase inversion using [EMIM]OAc-DMSO mixtures as solvent. Chem. Eng. Sci..

[27] Kuzin M.S., Skvortsov I.Y., Gerasimenko P.S., Subbotin A.V., Malkin A.Y. (2023). The role of the solvent nature in stretching polymer solutions (polyacrylonitrile spinning using different solvents). J. Mol. Liq..

[28] Makarov I.S., Golova L.K., Kuznetsova L.K., Antonov S.V., Kotsyuk A.V., Ignatenko V.Y., Kulichikhin V.G. (2016). Influence of Precipitation and Conditioning Baths on the Structure, Morphology, and Properties of Cellulose Films. Fibre Chem..

[29] Fink H.-P., Weigel P., Purz H.J. (2001). Structure formation of regenerated cellulose materials from NMMO-Solutions. Prog. Polym. Sci..

[30] Duolikun T., Ghazali N., Leo B.F., Lee H.V., Lai C.W., Johan M.R.B. (2020). Asymmetric Cellulosic Membranes: Current and Future Aspects. Symmetry.

[31] Makarov I.S., Golova L.K., Vinogradov M.I., Mironova M.V., Anokhina T.S., Arkharova N.A. (2021). Morphology and transport properties of membranes obtained by coagulation of cellulose solutions in isobutanol. Carbohydr. Polym..

[32] Brasier J. (1986). The Deceptively Versatile Non-Plastic. Mater. Des..

[33] Huang R.Y.M., Jarvis N.R. (1970). Separation of liquid mixtures by using polymer membranes. II. Permeation of aqueous alcohol solutions through cellophane and poly(vinyl alcohol). J. Appl. Polym. Sci..

[34] Nagy E., Borlai O., Ujhidy A. (1980). Membrane permeation of water—Alcohol binary mixtures. J. Membr. Sci..

[35] Ghosh I., Sanyal S.K., Mukherjea R.N. (1988). Pervaporation of methanol–ethylene glycol with cellophane membrane: Some mechanistic aspects. Ind. Eng. Chem. Res..

[36] Makarov I.S., Golova L.K., Bondarenko G.N., Anokhina T.S., Dmitrieva E.S., Levin I.S., Makhatova V.E., Galimova N.Z., Shambilova G.K. (2022). Structure, Morphology, and Permeability of Cellulose Films. Membranes.

[37] Makarov I.S., Budaeva V.V., Gismatulina Y.A., Kashcheyeva E.I., Zolotukhin V.N., Gorbatova P.A., Sakovich G.V., Vinogradov M.I., Palchikova E.E., Levin I.S. (2024). Preparation of Lyocell Fibers from Solutions of Miscanthus Cellulose. Polymers.

[38] Protz R., Lehmann A., Ganster J., Fink H.-P. (2021). Solubility and spinnability of cellulose-lignin blends in aqueous NMMO. Carbohydr. Polym..

[39] Byrne N., De Silva R., Ma Y., Sixta H., Hummel M. (2018). Enhanced stabilization of cellulose-lignin hybrid filaments for carbon fiber production. Cellulose.

[40] Ma Y., Stubb J., Kontro I., Nieminen K., Hummel M., Sixta H. (2018). Filament spinning of unbleached birch kraft pulps: Effect of pulping intensity on the processability and the fiber properties. Carbohydr. Polym..

[41] (1984). Cellulose. Method for Determination of Alpha Cellulose Content..

[42] Golova L.K. (2002). New Cellulose Fiber Lyocell. Russ. Chem. J..

[43] (1998). Sulfite Viscose Cellulose, Technical Specifications.

[44] Salem K.S., Kasera N.K., Rahman M.A., Jameel H., Habibi Y., Eichhorn S.J., Lucia L.A. (2023). Comparison and assessment of methods for cellulose crystallinity determination. Chem. Soc. Rev..

[45] Kostryukov S.G., Matyakubov H.B., Masterova Y.Y., Kozlov A.S., Pryanichnikova M.K., Pynenkov A.A., Khluchina N.A. (2023). Determination of Lignin, Cellulose, and Hemicellulose in Plant Materials by FTIR Spectroscopy. J. Anal. Chem..

[46] Makarov I., Palchikova E., Vinogradov M., Golubev Y., Legkov S., Gromovykh P., Makarov G., Arkharova N., Karimov D., Gainutdinov R. (2025). Characterization of Structure and Morphology of Cellulose Lyocell Microfibers Extracted from PAN Matrix. Polysaccharides.

[47] Baskakov S.A., Baskakova Y.V., Kabachkov E.N., Kichigina G.A., Kushch P.P., Kiryukhin D.P., Krasnikova S.S., Badamshina E.R., Vasil’ev S.G., Soldatenkov T.A. (2022). Cellulose from Annual Plants and Its Use for the Production of the Films Hydrophobized with Tetrafluoroethylene Telomers. Molecules.

[48] Graessley W.W. (2008). Polymeric Liquids and Networks: Dynamics and Rheology.

[49] Makarov I.S., Golova L.K., Kuznetsova L.K., Mironova M.V., Vinogradov M.I., Bermeshev M.V., Levin I.S., Kulichikhin V.G. (2019). Composite Fibers from Cellulose Solutions with Additives of Bis (Trimethylsilyl) Acetylene and Alkoxysilanes: Rheology, Structure and Properties. Fibre Chem..

[50] Kaplan D.L. (2013). Biopolymers from Renewable Resources.

[51] Nelson M.L., O’Connor R.T. (1964). Relation of certain infrared bands to cellulose crystallinity and crystal lattice type. Part I. Spectra of lattice types I, II, III and amorphous cellulose. J. Appl. Polym. Sci..

[52] Carrillo F., Colom X., Suñol J.J., Saurina J. (2004). Structural FTIR analysis and thermal characterization of lyocell and viscose-type fibres. Eur. Polym. J..

[53] Ornaghi H.L., Poletto M., Zattera A.J., Amico S.C. (2014). Correlation of the thermal stability and the decomposition kinetics of six different vegetal fibers. Cellulose.

[54] Bashar M.M., Zhu H., Yamamoto S., Mitsuishi M. (2017). Superhydrophobic surfaces with fluorinated cellulose nanofiber assemblies for oil–water separation. RSC Adv..

[55] Meng X., Dong Y., Zhao Y., Liang L. (2020). Preparation and modification of cellulose sponge and application of oil/water separation. RSC Adv..

[56] Dankovich T.A., Gray D.G. (2011). Contact Angle Measurements on Smooth Nanocrystalline Cellulose (I) Thin Films. JAST.

[57] Bruel C., Queffeulou S., Carreau P., Tavares J.R., Heuzey M.-C. (2020). Orienting Cellulose Nanocrystal Functionalities Tunes the Wettability of Water-Cast Films. Langmuir.

